# Current Trends in Venous Thromboprophylaxis for Inpatient Care

**DOI:** 10.3390/jpm16010018

**Published:** 2026-01-04

**Authors:** Maria Velliou, Vasiliki Bistola, John Parissis, Effie Polyzogopoulou

**Affiliations:** University Emergency Department, Attikon University Hospital, 12462 Athens, Greecevasobistola@med.uoa.gr (V.B.);

**Keywords:** venous thromboembolism, thromboprophylaxis, unfractionated heparin, low-molecular-weight heparin, fondaparinux, hospitalization

## Abstract

Thromboprophylaxis in hospitalized patients is a critical component of care aimed at preventing venous thromboembolism (VTE), a common and potentially fatal complication during hospitalization. The risk of VTE varies substantially across patient populations, influenced by the type of illness, including both surgical procedures and medical comorbidities, and requires individualized assessment. At the same time, the implementation of pharmacological thromboprophylaxis must carefully balance the risk of thrombosis against the potential for bleeding. Commonly used risk assessment models, such as the Padua and IMPROVE scores, can help clinicians stratify patients according to their individual risk of VTE and bleeding complications. The aim of the present review is to provide a structured synthesis of the current evidence on thromboprophylaxis strategies in hospitalized patients, critically appraise the performance and applicability of existing VTE and bleeding risk models and highlight how these tools can guide a tailored illness-specific approach to prophylactic decision-making. Where relevant, the review also outlines practical, risk-adapted algorithms to optimize thromboprophylaxis across diverse clinical settings.

## 1. Introduction

Venous thromboembolism (VTE), including deep vein thrombosis (DVT) and pulmonary embolism, is among the top five vascular diseases and the third most common cause of mortality. It is estimated to affect one million subjects per year in Europe and almost 10 million people globally, while its incidence seems to be much lower in Eastern countries [1,2,3]. The lifetime risk of VTE is 8% in the Western world, while about 20% of affected persons die within a year after diagnosis [1].

VTE is a multifactorial disease in which clinical conditions, pharmacologic treatments, genetic factors and demographic characteristics determine the individual’s thrombotic potential. Patients with cardiovascular disease, malignancies, fractures, lower limbs immobilization following trauma, heparin-induced thrombocytopenia, infection, COVID-19 disease, antiphospholipid antibody syndrome, autoimmune diseases, a previous history of VTE, obesity and a recent surgical procedure are at a greater risk for VTE [2].

Hospitalization due to an acute medical illness is also considered a triggering factor, even in the absence of immobilization. A previous observational cohort study has shown that hospital admission was associated with a 38-fold higher risk of VTE and the risk remained 3-fold greater up to three months after discharge [4]. Given that VTE is a major cause of morbidity and mortality, identification of patients at high VTE risk in the Emergency Department and initiation of thromboprophylaxis is an essential intervention to ensure patients’ safety during hospitalization.

Despite the well-recognized burden of VTE in hospitalized patients and the availability of validated risk assessment tools, their application in routine clinical practice remains inconsistent, and uncertainty persists regarding how best to integrate thrombotic and bleeding risks across diverse clinical scenarios. Variability in patient profiles, differences between surgical and medical conditions, and continually evolving evidence further complicate decision-making at the bedside. To address these challenges, the present review examines the widely adopted thrombotic and bleeding risk models used in hospitalized patients, evaluates their effectiveness in different clinical contexts, and synthesizes current evidence on optimal thromboprophylaxis strategies. It also aims to clarify how risk-adapted approaches can be applied in everyday clinical practice and to outline practical, evidence-based decision pathways.

## 2. Evidence on Thromboprophylaxis in Hospitalized Patients

Hospital-acquired VTE is a common and serious complication among acutely ill medical inpatients. Landmark trials, including the MEDENOX (Prophylaxis in Medical Patients with Enoxaparin), the PREVENT (Prospective Evaluation of Dalteparin Efficacy for Prevention of VTE in Immobilized Patients Trial) and the ARTEMIS (Arixtra for Thromboembolism Prevention in a Medical Indication Study), have demonstrated that pharmacologic thromboprophylaxis with low-molecular-weight heparin (LMWH) or fondaparinux significantly reduces the VTE risk in this population (Table 1) [5,6,7].

The MEDENOX study included 1102 hospitalized patients aged > 40 years that were randomly assigned to receive enoxaparin 40 mg, enoxaparin 20 mg or placebo once daily for 6–14 days. The study showed that the incidence of VTE was significantly lower in the group of enoxaparin 40 mg compared to the placebo group (5.5% versus 14.9%). Importantly, there was no significant difference in the incidence of VTE between the groups, showing that enoxaparin 40 mg is effective in preventing VTE without increasing serious bleeding risk [5].

Similarly, the PREVENT study was a randomized, double-blind, placebo-controlled, multicenter trial of 3706 patients aged ≥ 40 years admitted to the hospital due to an acute medical condition (acute heart failure, acute respiratory failure, infection, rheumatologic disorder, inflammatory bowel disease) who were expected to be hospitalized for ≥4 days and remain in bed for ≥3 days, with at least one risk factor for VTE. The patients were randomly assigned to receive either dalteparin or placebo for 14 days and were followed for 90 days. The primary endpoint was the combination of symptomatic DVT, symptomatic pulmonary embolism, asymptomatic proximal DVT detected by ultrasound at day 21 and sudden death by day 21. The study demonstrated that thromboprophylaxis with dalteparin the primary endpoint reduced by 45% and this effect was maintained by day 90 with the incidence of symptomatic VTE being 0.93% in the dalteparin group and 1.33% in the placebo group. By day 21, no patient in the dalteparin group and two patients in the placebo group had a fatal pulmonary embolism. Regarding safety outcomes, while minor bleeding events were slightly more frequent with dalteparin, rates of major and fatal bleeding remained very low, confirming that dalteparin effectively prevents VTE in medically ill, immobilized patients with minimal bleeding risk (fatal bleeding: 0.11% versus 0.05%, major bleeding: 0.49% versus 0.16%; minor bleeding: 1.03% versus 0.55%) [6].

The ARTEMIS trial included 849 patients aged ≥ 60 years that were hospitalized due to congestive heart failure, acute respiratory illness, acute infectious disease or acute inflammatory disease and had ≥4 days of immobilization. The patients were randomly assigned to receive either fondaparinux or placebo for 6–14 days and were followed for one month. The study showed that fondaparinux reduced the risk of VTE on venography and symptomatic VTE by 46.7%. By day 15, the incidence of VTE was 5.6% among patients who received fondaparinux compared to 10.5% among those received placebo. No difference in the frequency of major bleeding episodes was recorded between the two groups. This demonstrates that fondaparinux provides substantial protection against VTE while maintaining a favorable safety profile [7].

Overall, these trials collectively show that thromboprophylaxis with LMWH or fondaparinux is both effective and safe in acutely ill medical inpatients, particularly among older, immobilized or otherwise high-risk patients.

Beyond traditional parenteral agents, several large, randomized trials have investigated extended-duration thromboprophylaxis with direct oral anticoagulants (DOACs) in this patient population [8,9,10]. The ADOPT study evaluated extended thromboprophylaxis using oral apixaban 2.5 mg twice daily for 30 days versus standard enoxaparin 40 mg for 6–14 days in acutely ill patients hospitalized for at least three days with congestive heart failure, respiratory failure or other medical conditions, and who had at least one additional risk factor for VTE. The study found that the prolonged apixaban regimen did not demonstrate superior efficacy; the primary composite VTE outcome occurred in 2.71% of patients receiving apixaban compared with 3.05% of those receiving enoxaparin. Additionally, apixaban was linked to a higher rate of major bleeding events (0.47% versus 0.19%) [8].

The MAGELLAN trial enrolled 8101 patients who had been hospitalized within 72 h for an acute medical illness and had reduced mobility. Participants were randomly assigned to receive oral rivaroxaban 10 mg once daily for 35 ± 4 days or subcutaneous enoxaparin 40 mg once daily for 10 ± 4 days during hospitalization. At day 35, rivaroxaban significantly reduced the composite outcome of DVT (asymptomatic or symptomatic), symptomatic pulmonary embolism, or VTE-related death (4.4% versus 5.7% with enoxaparin), demonstrating superiority over short-term prophylaxis. However, this benefit was accompanied by a notably higher risk of clinically significant bleeding (4.1% versus 1.7%) [9].

Last but not least, the APEX trial investigated extended thromboprophylaxis with the oral factor Xa inhibitor betrixaban (80 mg once daily for 35–42 days) versus a standard shorter course of enoxaparin (40 mg for 10 ± 4 days) in patients at high risk for VTE, such as those with elevated d-dimer levels or aged 75 years and older. In the overall study population, betrixaban lowered the incidence of asymptomatic proximal DVT or symptomatic VTE compared with enoxaparin (5.3% versus 7.0%). Notably, the rates of major bleeding were comparable between the two treatment groups (0.7% versus 0.6%) [10].

## 3. VTE and Bleeding Risk Stratification

**Figure 1:**
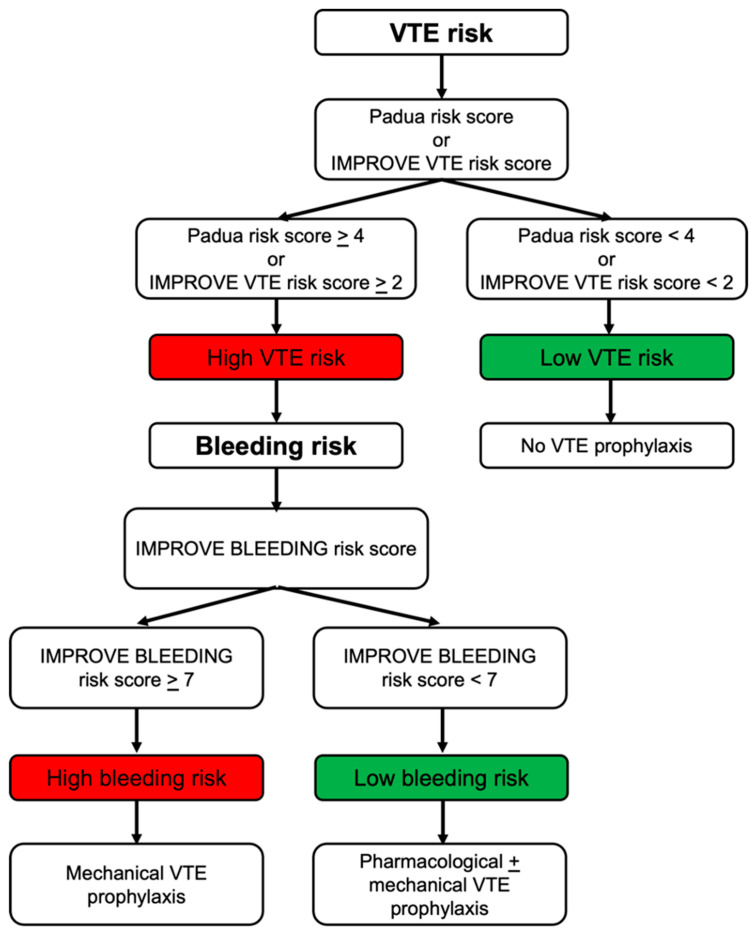
An individualized stepwise approach on thromboembolic prophylaxis in hospitalized patients. Abbreviation: VTE: venous thromboembolism.

The American Society of Hematology (ASH) has endorsed the use of risk assessment models (RAMs) as an approach to simplify VTE and bleeding risk stratification in medical inpatients and guide provision of thromboprophylaxis to those at high risk (Figure 1) [11,12]. However, despite existing guidelines [12], pharmacologic thromboprophylaxis is often either underused in high-risk patients or overused in low-risk patients. A Swiss prospective cohort study of 1352 hospitalized patients showed that 36.7% to 41.3% were categorized as high VTE risk but did not receive anticoagulants, while 37.2% to 47.6% were characterized as low VTE risk and received thromboprophylaxis, highlighting a gap between guideline recommendations and clinical practice [13]. Potential causes of underuse include limited awareness of current guidelines, uncertainty regarding individual bleeding risk, variability in clinician experience and lack of standardized institutional protocols [13,14]. Conversely, overuse may result from automated order sets that do not integrate individualized risk assessments, defensive prescribing practices or misinterpretation of VTE risk in low-risk patients [13,15,16]. Institutional protocols that mandate systematic risk assessment at admission and prior to prescribing anticoagulants can standardize care, improve adherence and reduce variability in clinical practice. These challenges highlight the importance of structured risk assessment tools and consistent clinical workflows to optimize prophylaxis.

**VTE risk scores.** The Padua VTE RAM and the IMPROVE VTE RAM represent the most extensively studied validated risk scores for the identification of hospitalized patients at a greater risk for VTE. The parameters that are included in the Padua VTE RAM are: active cancer, previous VTE, reduced mobility, known thrombotic condition, recent trauma/surgery, trauma or stroke in the previous month, age > 70 years, heart or respiratory failure, acute myocardial infraction or stroke, acute infection or rheumatologic disorder, body mass index (BMI) > 30 kg/m^2^ and use of hormone replacement therapy. A score of 4 or more indicates a greater VTE risk and thromboprophylaxis is recommended. The IMPROVE VTE RAM takes into account factors like previous VTE, known thrombophilia, current lower limb paralysis, active cancer, immobilization for more than seven days, age > 60 years and admission at ICU (Intensive Care Unit) or CCU (Coronary Care Unit). A score of 2 or more indicates inpatients at higher risk for VTE [12]. These tools allow clinicians to identify high-risk inpatients more accurately and target prophylaxis effectively. They can be integrated into electronic medical records (EMRs) to automatically calculate patient scores in the Emergency Department, incorporated into standardized order sets for thromboprophylaxis or included in bedside checklists for real-time risk assessment [17].

D-dimer, a fibrin degradation product, has also been recognized as a predictive factor of increased VTE risk in hospitalized patients. The MAGELLAN trial found that the incidence of VTE was 3.5-fold higher in patients with baseline d−dimer of greater than 2× the upper limit of normal (ULN). High d-dimer concentrations had similarly strong associations to already established risk factors for VTE, such as malignancy and advanced age [18]. The incorporation of d-dimer levels to the IMPROVE VTE risk score, thereby creating the modified version IMPROVEDD VTE score, was shown to improve VTE risk stratification in hospitalized patients [19]. Measuring this biomarker at admission can, thus, be combined with RAMs in EMRs or bedside checklists to further refine thromboprophylaxis decisions.

For oncology patients, the Khorana score provides validated assessment. The main parameters of the score are: stomach or pancreatic malignancy (2 points); lymphoma, lung, gynecologic, bladder or testicular cancer (1 point); pre-chemotherapy platelet count ≥ 350 × 10^9^/L (1 point); pre-chemotherapy hemoglobin level < 10 g/dL or use of RBC (red blood cell) growth factors (1 point); pre-chemotherapy leukocyte count > 11 × 10^9^/L (1 point) and BMI ≥ 35 kg/m^2^ (1 point). The Khorana score classifies cancer patients into low (0 points), intermediate (1–2 points) or high (≥3 points) VTE risk [20]. Embedding this score into electronic oncology order sets allows automatic identification of high-risk patients for prophylaxis while avoiding unnecessary anticoagulation in low-risk patients.

In surgical patients, the Caprini VTE score guides thromboprophylaxis decisions by accounting 40 distinct individual VTE risk factors, such as type of surgery, and personal and family history, and distinguishes patients at low (score: 0–1), moderate (score: 2), high (score: 3–4) or very high (score ≥ 5) risk [21,22]. This comprehensive approach allows precise identification of surgical patients who may benefit most from prophylaxis. Integration into preoperative EMR workflows and perioperative checklists can ensure that high-risk surgical patients receive timely prophylaxis.

Table 2 summarizes the currently available VTE risk scores used in hospitalized patients.

**Bleeding risk score.** Even though thromboprophylaxis reduces the incidence of hospital acquired VTE, bleeding remains the most important complication in patients treated with anticoagulants. The IMPROVE (International Medical Prevention Registry on VTE) multinational, observational study demonstrated that the incidence of major and non-major bleeding within 14 days of admission was 3.2%. Active gastroduodenal ulcer, a prior bleeding event and low platelet count were the strongest independent risk factors for bleeding during hospitalization [23]. Likewise, a prospective, observational study from the Netherlands showed that the prevalence of in-hospital bleeding events among anticoagulant users was 7.2%, with the vast majority (78.5%) categorized as major bleeding. The median length of stay was 18 (8.5–34.5) days in patients with a bleeding event and 7 (4–13) days among those without a bleeding event. Female gender, surgical procedures (high or low bleeding risk procedures) and non-surgical interventions (endoscopic interventions, endovascular coiling) were identified as risk factors [24].

The IMPROVE bleeding RAM is a validated risk score aiming to identify in-hospital patients at higher risk for bleeding. It includes 13 clinical and laboratory factors: moderate (GFR 30–59 mL/min/m^2^) or severe (GFR < 30 mL/min/m^2^) renal failure, male gender, age 40–84 years or ≥85 years, rheumatic disease, active malignancy, central venous catheter placement, ICU or CCU admission, liver failure (INR > 1.5), platelet count < 50 × 10^9^/L, prior bleeding within the last three months before admission and active gastric or duodenal ulcer (Table 3). A score of 7 or more indicates patients at greater bleeding risk and should be taken into account for designing a safe and individualized thromboprophylaxis plan [12].

Integrating RAMs into routine practice has a substantial impact on healthcare resources. Accurate risk stratification reduces unnecessary use of anticoagulants, lowers the incidence of bleeding complications, optimizes pharmacy and nursing workload, and can shorten hospital stays, reducing overall costs [25,26,27]. Systematic implementation also enhances quality of care, ensuring evidence-based, standardized prophylaxis decisions, improving guideline adherence, reducing preventable complications, and supporting institutional performance metrics [25,28].

By combining VTE and bleeding RAMs, along with laboratory markers such as d-dimer, into electronic systems, standardized order sets and bedside checklists, clinicians can implement risk-adapted thromboprophylaxis efficiently, improving patient safety and adherence to guidelines.

## 4. Recommendations in Specific Clinical Conditions

**Heart failure.** Patients with heart failure experience a greater risk for VTE, even when in sinus rhythm, mainly owing to static blood flow, hypercoagulability and endothelial dysfunction [29]. Notably, the risk is reversely correlated to left ventricular ejection fraction (LVEF). A case-control study demonstrated that a LVEF < 20% was correlated with a 38.3-fold increased risk of thromboembolic events [30]. Similarly, an analysis of the SCD-HeFT (Sudden Cardiac Death in Heart Failure Trial) showed that reduced LVEF was a significant predictor of VTE among 2114 patients without a known history of atrial fibrillation or atrial flutter. On the other hand, for every 5% rise of the LVEF, the risk of VTE decreased by 18% [31]. Hence, pharmacological prophylaxis with LMWH, fondaparinux or low-dose unfractionated heparin (UFH) is recommended in all hospitalized patients with heart failure who are not already receiving anticoagulation, unless they are at high bleeding risk [32,33].

**Cancer.** Malignancy is correlated with a fivefold greater hazard of VTE with the absolute risk ranging between 2% and 17% during hospitalization [34]. The risk is higher in several types of tumors, such as pancreatic, gastric, lung and primary brain cancer [35]. Therefore, thromboprophylaxis in oncological patients during hospitalization remains an important issue.

**Figure 2:**
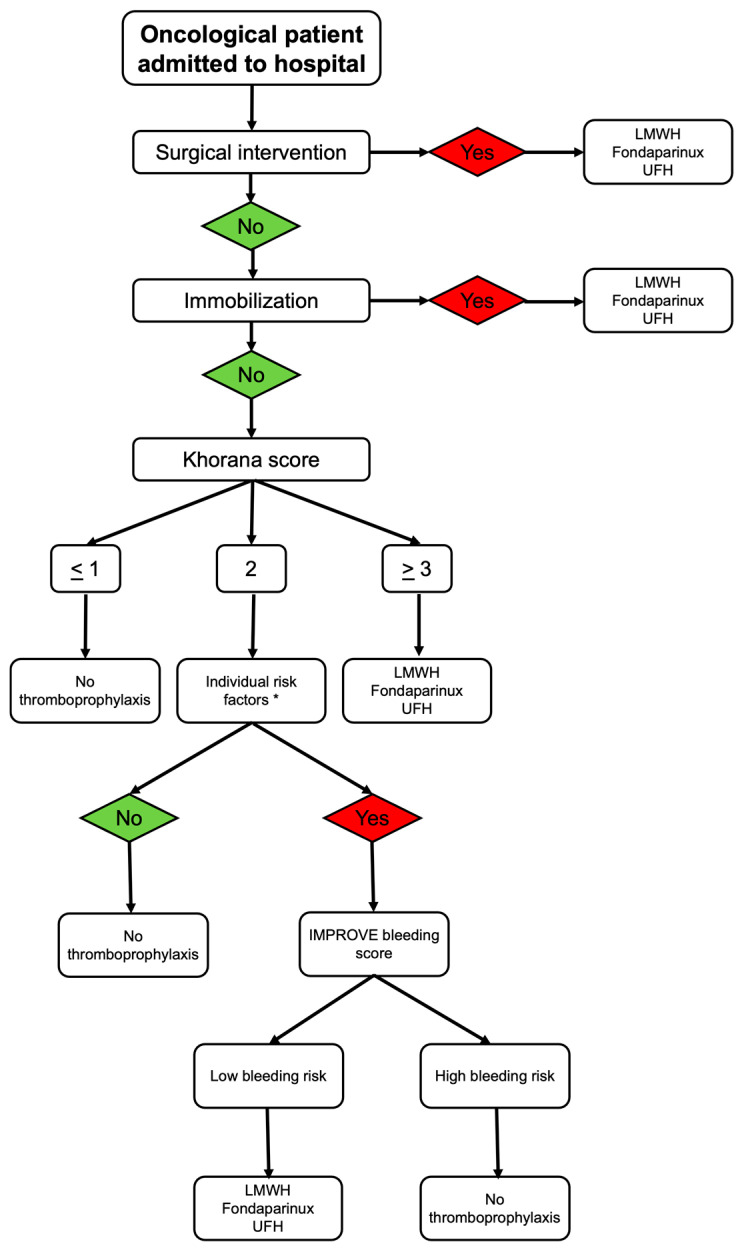
A proposed algorithm for thromboprophylaxis in hospitalized cancer patients. * immobilization at home, thrombogenic therapy, history of VTE, hereditary thrombophilia. Abbreviations: low-molecular-weight heparin (LMWH); unfractionated heparin (UFH); VTE: venous thromboprophylaxis.

The American Society of Clinical Oncology (ASCO) [36], the European Society of Medical Oncology (ESMO) [35] and the ASH [37] have published guidelines on thromboprophylaxis among cancer inpatients. For all immobilized patients with hematologic or solid malignancies admitted to the hospital due to an acute medical illness or a planned surgical intervention, thromboprophylaxis with LMWH, fondaparinux or UFH is recommended. In non-immobilized patients, the Khorana score should be calculated and those with a score of ≤1 should not receive thromboprophylaxis, while those with a score of ≥3 are candidates for thromboprophylaxis with LMWH, fondaparinux or UFH. In patients with a Khorana score of 2 and no additional individual risk factors for VTE, including immobilization at home, thrombogenic therapy, history of VTE and hereditary thrombophilia, thromboprophylaxis is not indicated. On the other hand, in patients with a Khorana score of 2 and at least one of the aforementioned individual risk factors for VTE, thromboprophylaxis should be considered based on the presence or absence of risk factors for bleeding, such as severe thrombocytopenia, susceptibility to falls or history of bleeding. In the presence of bleeding risk factors, thromboprophylaxis is contraindicated; otherwise, thromboprophylaxis is suggested (Figure 2) [35,36,37].

**Surgery.** VTE is considered a serious complication in surgical patients receiving general anesthesia during hospitalization and it seems that the risk remains elevated up to two months following discharge [38,39]. Procedure type and duration, infectious complications and patient-specific factors, such as age, comorbidities and a personal history of VTE, play a crucial role in postoperative VTE risk [21].

The ASH recommends both mechanical and pharmacological thromboprophylaxis in patients at high VTE risk and suggests mechanical methods over pharmacological thromboprophylaxis in patients at high bleeding risk. In cases of mechanical thromboprophylaxis, intermittent compression devices are indicated over graduated compression stockings [40].

Major lower-extremity orthopedic surgeries, including total or partial knee or hip arthroplasty surgery and hip fracture repair, carry a greater risk for VTE compared to other orthopedic procedures, with the estimated incidence being approximately 4% [41]. Thus, thromboprophylaxis with aspirin or anticoagulants is suggested for patients who have undergone hip or knee arthroplasty. When anticoagulants are used, DOACs, LMWH or fondaparinux are recommended over warfarin and UFH. For patients after hip fracture repair, thromboprophylaxis with LMWH, fondaparinux or UFH is suggested [40,42].

**Figure 3:**
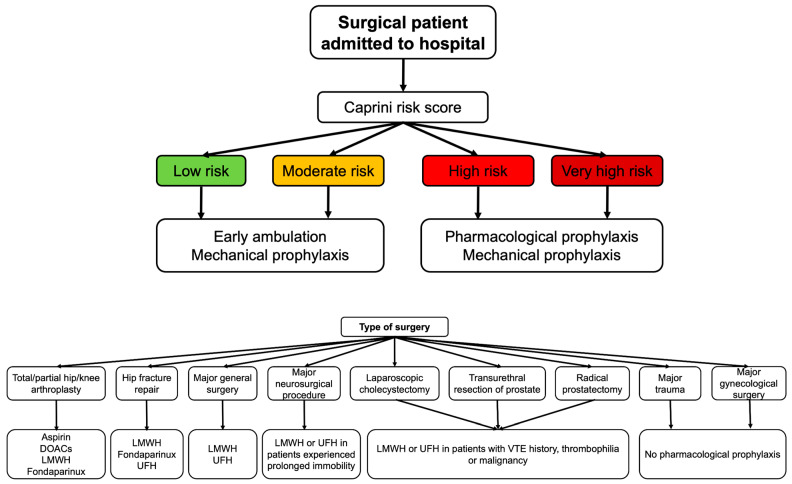
Thromboprophylaxis in hospitalized patients who have undergone surgical procedures. Abbreviations: DOACs: direct oral anticoagulants; LMWH: low-molecular-weight heparin; UFH: unfractionated heparin; VTE: venous thromboprophylaxis.

Pharmacological thromboprophylaxis with LMWH or UFH is also preferred in patients who have undergone a major general surgery. Likewise, pharmacological prophylaxis with LMWH over UFH is recommended only in patients who have undergone major neurosurgical procedures and experiencing prolonged immobility following surgery. On the other hand, the ASH guidelines suggests against pharmacological prophylaxis in patients who have undergone laparoscopic cholecystectomy, transurethral resection of the prostate or radical prostatectomy, excluding those with known risk factors for VTE, such as a history of VTE, thrombophilia and malignancy. For those experiencing major trauma or who have undergone a major gynecological surgery, no pharmacological thromboprophylaxis is suggested [40]. Figure 3 summarizes current recommendations on thromboprophylaxis in surgical patients admitted to the hospital.

**Stroke.** VTE represents a common complication among immobile hospitalized patients with acute ischemic stroke [43]. However, these patients are at increased risk of hemorrhagic transformation [44] and managing thromboprophylaxis in this setting is a key challenge. The European Stroke Organization (ESO) advises intermittent pneumatic compression (thigh-length, sequential) combined with pharmacological prophylaxis, namely UFH, LMWH or heparinoid, for immobile patients hospitalized due to acute ischemic stroke [45]. Fondaparinux represents an alternative option since it seems to be as safe as UFH for VTE prophylaxis in this population. An analysis of 644 consecutive patients with acute ischemic stroke that were randomly assigned to receive either fondaparinux or UFH showed that major hemorrhages were less common in the fondaparinux group (1.2%) compared to the UFH group (3.7%), but this difference was not statistically significant [46].

VTE is also a serious concern in patients with acute non-traumatic intracerebral hemorrhage, affecting approximately 7% [47]. Meanwhile, the risk of DVT is four times greater than in those with acute ischemic stroke [48]. The American Heart Association (AHA) and the American Stroke Association suggest intermittent pneumatic compression from the day of diagnosis and initiating low-dose UFH or LMWH in order to reduce the risk for pulmonary embolism at 24–48 h from ICH onset in non-ambulatory patients [49].

**Sepsis/septic shock.** Septic patients are invariably affected by activation of coagulation and are at high risk for VTE. A study of approximately five million admissions with the diagnosis of sepsis (sepsis without shock: 4,050,824 hospitalizations, septic shock: 968,545) showed that the incidence of pulmonary embolism was 1.2% among those with sepsis and 2.3% among those with septic shock [50]. Another study of 918 hospitalized septic patients demonstrated that the incidence of DVT was 23.4% [51]. The Surviving Sepsis Campaign (SSG) guidelines recommend the use of pharmacological VTE prophylaxis with LMWH over UFH in hospitalized patients with sepsis or septic shock for VTE prevention [52].

**Chronic kidney disease.** Available evidence indicates that chronic kidney disease (CKD) is associated with hypercoagulability, increasing the risk of VTE in this population [53]. A large community-based study that included about 19,000 middle-aged and elderly adults demonstrated that the relative VTE risk was 1.28 among those with mild renal impairment and 2.09 among those with stage 3/4 CKD after adjustment for age, gender, race and center compared to those with normal kidney function [54]. Paradoxically, CKD is correlated with a 35% higher risk for bleeding [55]. Given that both thrombotic and bleeding risk are elevated in patients with CKD, the clinical decision on thromboprophylaxis is challenging. The National Institute for Health and Care Excellence (NICE) suggests to balance the individual VTE and bleeding risk and, if indicated, LMWH or UFH should be given in a reduced dose [56].

Table 4 summarizes the recommended thromboprophylaxis regimens for hospitalized patients across specific clinical conditions.

## 5. Limitations and Future Directions

Despite extensive research supporting thromboprophylaxis in hospitalized patients, several evidence gaps remain. The predictive performance and generalizability of existing RAMs, such as the Padua, IMPROVE, Caprini and Khorana scores, require further validation across diverse populations, including elderly, multi-morbid and renal-impaired patients [57]. Implementation strategies for integrating RAMs into clinical workflows, electronic health records and standardized order sets are not fully established [58], and clinician adherence to risk-guided prophylaxis remains variable [59].

It is important to emphasize that no risk-assessment tool is perfect, and clinical judgment should remain central to decision-making. While RAMs provide structured guidance, they cannot account for all factors influencing an individual patient’s thrombotic risk. These scores should be interpreted within the context of each patient’s overall clinical condition, with the multidisciplinary team weighing the potential benefits and risks of anticoagulation. Engaging patients in shared decision-making is essential to ensure they understand their personalized risk–benefit profile before initiating prophylaxis. In this way, RAMs serve as a supportive tool rather than a substitute for careful, individualized clinical evaluation.

In addition, economic and patient-centered outcomes warrant further investigation. Evidence on the cost-effectiveness of RAM-guided prophylaxis, including impacts on hospital stay, drug utilization and overall healthcare resources, remains limited. Furthermore, few studies have assessed long-term patient-centered outcomes, such as quality of life, functional recovery or patient preferences. Evidence gaps also persist in special populations, including patients with obesity, sepsis or multiple comorbidities, who are often underrepresented in clinical trials. Addressing these gaps is crucial to optimizing individualized thromboprophylaxis strategies and informing future research and clinical practice.

## 6. Conclusions

Thromboprophylaxis plays a critical role in preventing VTE among hospitalized patients and an individualized approach is essential. The clinical decision to initiate thromboprophylaxis during hospitalization involves assessing various factors, such as the patient’s thrombotic and bleeding risk and the type of medical condition or surgery. The main pharmacologic options for thromboprophylaxis include LMWH, UFH and fondaparinux. Pharmacological thromboprophylaxis is recommended in all hospitalized patients with heart failure, malignancy or sepsis/septic shock, unless they are at high bleeding risk. Patients who have undergone a major lower-extremity orthopedic surgery or a major general surgery are candidates for both mechanical and pharmacological thromboprophylaxis. Thromboprophylaxis is indicated for immobile patients hospitalized due to acute ischemic stroke. In non-ambulatory patients with ICH, intermittent pneumatic compression is recommended from the day of diagnosis while low-dose UFH or LMWH should be initiated 24–48 h from symptom onset. Anticoagulants in reduced dose are suggested in hospitalized patients with CKD.

## Figures and Tables

**Table 1:** Landmark trials that evaluated the VTE risk in acutely ill medical patients during hospitalization.

	MEDENOX [5], 1999	PREVENT [6], 2004	ARTEMIS [7], 2006	ADOPT [8], 2011	MAGELLAN [9], 2013	APEX [10], 2016
**Patients (*n*)**	1102	3706	849	6528	8101	7513
**Age (years)**	>40	>40	>60	>40	>40	>40
**Anticoagulant**	Enoxaparin 20 mg or 40 mg for 7 days	Dalteparin 5000 IU for 14 days	Fondaparinux 2.5 mg for 6–14 days	Apixaban 2.5 mg for 30 days	Rivaroxaban 10 mg for 35 ± 4 days	Betrixaban 80 mg for 35–42 days
**Comparator**	Placebo	Placebo	Placebo	Enoxaparin 40 mg for 6–14 days	Enoxaparin 40 mg for 10 ± 4 days	Enoxaparin 40 mg for 10 ± 4 days
**Follow-up**	14 days	90 days	30 days	30 days	35 ± 4 days	47 days
**Outcomes**	VTE risk (5.5% vs. 14.9%) RR 0.37 (95%CI: 0.21–0.64)	VTE/sudden death risk (2.77% vs. 4.96%) RR: 0.55 (95%CI: 0.38–0.80)	VTE risk (5.6% vs. 10.5%) RR: 0.54 (95%CI: 0.31–0.92)	No superiority (2.71% vs. 3.05%) RR: 0.87 (95%CI: 0.62–1.23)	VTE risk (4.4% vs. 5.7%) RR: 0.77 (95%CI: 0.62–0.96)	VTE risk (5.3% vs. 7%) RR: 0.76 (95%CI: 0.63–0.92)

Abbreviations: RR: relative risk; VTE: venous thromboembolism.

**Table 2:** Clinical risk scores for predicting VTE in hospitalized patients.

VTE Risk Score	Population	Risk Determinants	VTE Prophylaxis Eligibility Thresholds
**Padua VTE RAM**	Acutely ill medical inpatients	Active cancer, previous VTE, reduced mobility, thrombophilia, recent trauma/surgery, elderly age, heart/respiratory failure, acute MI/stroke, acute infection/rheumatologic disorder, obesity, hormone therapy	≥4: high risk
**IMPROVE VTE RAM**	Acutely ill medical inpatients	Previous VTE, known thrombophilia, active cancer, immobilization ≥ 7 days, ICU/CCU admission, age > 60, lower-limb paralysis	≥2: high risk
**IMPROVE-DD VTE Score**	Acutely ill medical inpatients	Same as IMPROVE + elevated D-dimer (≥2× ULN)	≥2: high risk
**Khorana Score**	Oncology patients	Cancer type, platelet count, hemoglobin or RBC growth factors use, leukocyte count, BMI ≥ 35	0: low risk 1–2: intermediate risk ≥3: high risk
**Caprini VTE Score**	Surgical patients	40 distinct individual VTE risk factors including age, BMI > 25, cancer, history of VTE, thrombophilia, immobilization, surgery type, pregnancy, hormonal therapy, varicose veins, edema, inflammatory conditions	0–1: low risk 2: moderate risk 3–4: high risk ≥5: very high risk

Abbreviations: BMI: body mass index; CCU: Coronary Care Unit; ICU: Intensive Care Unit; RAM: risk assessment models; RBC: red blood cells; VTE: venous thromboembolism.

**Table 3:** IMPROVE bleeding risk score in hospitalized patients.

Predictor	Points
Active gastric or duodenal ulcer	4.5
History of bleeding within 3 months before admission	4
Platelet count < 50 × 10^9^/L	4
Age 40–84 years	1.5
Age ≥ 85 years	3.5
Hepatic failure (INR > 1.5)	2.5
Moderate renal failure (GFR 30–59 mL/min)	1
Severe renal failure (GFR < 30 mL/min)	2.5
ICU or CCU admission	2.5
Central venous catheter placement	2
Rheumatic disease	2
Active cancer	2
Male sex	1

Abbreviations: CCU: Coronary Care Unit; GFR: glomerular filtration rate; ICU: Intensive Care Unit.

**Table 4:** Recommended thromboprophylaxis regimens for inpatients in specific clinical settings.

Clinical Condition	Recommended Thromboprophylaxis Regimens
Heart failure [32,33]	LMWH, fondaparinux, or low-dose UFH
Cancer [35,36,37]	LMWH, fondaparinux, or UFH
Major general surgery [40]	LMWH or UFH
Hip or knee arthroplasty [40,42]	LMWH, fondaparinux, DOACs, or aspirin
Hip fracture repair [40,42]	LMWH, fondaparinux, UFH
Major neurosurgery [40]	LMWH preferred over UFH
Acute ischemic stroke [45,46]	Intermittent pneumatic compression + LMWH, UFH, fondaparinux or heparinoid
Intracerebral hemorrhage [49]	Intermittent pneumatic compression + low-dose UFH or LMWH
Sepsis/septic shock [52]	LMWH preferred over UFH
Chronic kidney disease [56]	LMWH or UFH (dose-adjusted)

Abbreviations: DOACs: direct oral anticoagulants; UFH: unfractionated heparin; LMWH: low-molecular-weight heparin.

## Data Availability

No new data were created or analyzed in this study. Data sharing is not applicable to this article.
